# Widely Viewed English Language YouTube Videos Relating to Diabetic Retinopathy: A Cross-Sectional Study

**DOI:** 10.2196/diabetes.6450

**Published:** 2016-12-14

**Authors:** Corey Hannah Basch, Isaac Chun-Hai Fung, Alyssa Berdnik, Charles E Basch

**Affiliations:** 1 Department of Public Health William Paterson University Wayne, NJ United States; 2 Department of Epidemiology Jiann-Ping Hsu College of Public Health Georgia Southern University Statesboro, GA United States; 3 Teachers College Columbia University New York, NY United States

**Keywords:** diabetic retinopathy, social media, YouTube

## Abstract

**Background:**

An emergent source of information on health issues is the Internet. One such platform with 1 billion users is YouTube, the global video-sharing service.

**Objective:**

The purpose of this study was to describe the content and characteristics of the most widely viewed YouTube videos related to diabetic retinopathy.

**Methods:**

Videos were sorted according to number of views using the key words “diabetic retinopathy.” For each video, general descriptive information was collected. This information included date and source of upload (news, professional, or consumer), length, and total number of views as of July 18, 2016. Content categories were largely informed by a National Eye Institute fact sheet. Each video was viewed to determine which, if any, of the given content categories were present.

**Results:**

Of the 98 most widely viewed videos related to diabetic retinopathy, 42 were generated by consumers, 40 were generated by professionals, and 16 were generated from news-based sources. The largest number of views were generated from professionals (624,770/994,494, 63.82%). Compared with professional videos, consumer videos were viewed less frequently (*W*=622, *P*=.04). The main purpose of the majority of videos was to provide information (59/98, 60%), and most of the videos showed or mentioned retinopathy in general (75/98, 77%). Smaller numbers offered information about specific types of retinopathy, namely proliferative (26/98, 27%) and nonproliferative (17/98, 17%). Compared with consumer-generated videos, professional videos were 5.57 times more likely to mention that diabetic retinopathy can go unnoticed (95% CI 1.59-26.15). More than 80% (80/98) of the most widely viewed videos did not address the asymptomatic nature of the disease, only about one-third (33/98) mentioned prevention, and only 58 of the 98 videos (59%) mentioned screening.

**Conclusion:**

Future research is needed to identify aspects of YouTube videos that attract viewer attention and best practices for using this medium to increase diabetic retinopathy screening among people with diabetes.

## Introduction

Diabetic retinopathy is the leading cause of blindness in adults of working age in the United States [1]. Almost 1 in 3 individuals aged 40 years and older with diabetes in the United States (28.5%) is afflicted with diabetic retinopathy or vision-threatening diabetic retinopathy [2], and rates are expected to triple between 2005 and 2050 [3]. Compared with non-Hispanic whites, the crude prevalence for both diabetic retinopathy and vision-threatening diabetic retinopathy is significantly higher for non-Hispanic blacks [2]. Duration of disease is associated with increased risk for developing diabetic retinopathy [1]. The visual impairment or blindness caused by diabetic retinopathy can be delayed or prevented through screening that results in early detection and, when appropriate, treatment with laser photocoagulation of retinal blood vessels. But while the natural history of the disease and how it can be prevented or minimized has been known for decades, only about 60% of people with diabetes receive an annual screening [4]. Rates of diabetic retinopathy screening have been shown to be higher among non-Hispanic whites than ethnic/racial minority groups [5].

People with diabetes are turning to the Internet for information. A study of young adults with diabetes indicated that they frequent websites uploaded by both professionals and consumers [6]. A recent literature review suggested that social media had a positive impact on chronic disease care [7]. In a study of diabetes-related Facebook pages, facets of social media that may have a positive influence on health promotion were examined [8]. Video-sharing platforms offer an array of information ranging from personal experiences to clinical advice on disease management [9], yet we did not identify any published studies on the nature of the most widely viewed YouTube videos on diabetic retinopathy. The purpose of this study was, therefore, to describe the source, content, and selected characteristics of the most widely viewed YouTube videos on this largely preventable disease that causes a substantial burden of vision loss.

## Methods

### Background

Videos were searched on YouTube.com using Chrome as a browser with a clean search history. The search term “diabetic retinopathy” was used for this study. Video popularity was established by filtering videos by total view count. The cut point of 100 most popular videos was set, and 2 of the videos were excluded because they were not in English. Thus, the final sample included 98 videos. The National Eye Institute (NEI) fact sheet entitled “Facts About Diabetic Eye Disease” was used as a guide in creating categories to code the content of the videos [1]. In addition, categories were added deductively. At the time the categories were created, the NEI fact sheet had been reviewed in September 2015. For each video, general descriptive information was collected. This information included source of the upload, date of upload, length, and total number of views as of July 18, 2016.

Consumer videos were defined as those uploaded by a user with no depicted professional affiliations. Professional videos were defined as those posted by a trained health professional. News clips included any news from a television network or Internet-based news station. One author (AB) coded the entire sample of 100 videos. To demonstrate interrater reliability, 10 videos were chosen using a random number generator and were then coded by both AB and CHB. For the 10 videos that were doubly coded, Cohen’s kappa was .8 and percentage agreement was 90% for one category (“Purpose of the video was to provide information about diabetic retinopathy”); for all other categories, there was 100% agreement.

Content categories were coded as “yes—mentioned” or “no—not mentioned” for each topic category. The categories used to code the videos were as follows: (1) gender of person providing information in the video (4 categories: no people shown, men shown, women shown, and both men and women shown), (2) purpose of the video was to provide information about diabetic retinopathy, (3) showed or mentioned diabetic retinopathy, (4) showed or mentioned proliferative diabetic retinopathy, (4) showed or mentioned nonproliferative diabetic retinopathy, (5) mentioned screening for diabetic retinopathy, (6) mentioned macular degeneration, (7) mentioned vision loss or blindness, (8) mentioned cataracts, (9) mentioned pain (if any) associated with diabetic retinopathy, (10) mentioned anxiety or fear of the diagnosis or screening, (11) mentioned control of diabetes, (12) mentioned symptoms (if any) for diabetic retinopathy, (13) mentioned treatment (if any) for diabetic retinopathy, (14) mentioned prevention (if any) for diabetic retinopathy, (15) mentioned that diabetic retinopathy can go unnoticed, and (16) mentioned retinal detachment.

### Statistical Analysis

Analysis was conducted using R version 3.3.0 (The R Foundation). Descriptive statistics were obtained using functions “summ” and “ci” from R package epiDisplay version 3.2.2.0 [10]. Wilcoxon rank-sum test was performed for pairwise comparison on views and lengths of videos between the 3 categories, given that their distributions were not normal. The correlation between the lengths of the videos and their number of views was assessed using Spearman’s rank order correlation coefficient. Logistic regression models were applied when the outcome variable was binary. In the case of gender of person providing information in the videos, where the variable was ordinal with 4 categories, multinomial logistic regression models were applied using the R package mlogit version 0.2-4 [11].

### Ethical Approval

The institutional review boards at William Paterson University and Teachers College, Columbia University, do not review studies that do not involve human subjects.

## Results

Descriptive statistics for the videos are presented in Table 1. Of the 98 widely viewed videos related to diabetic retinopathy, 42 were generated by consumers, 40 were generated by professionals, and 16 were generated from news-based news sources. Collectively, these videos were viewed almost 1 million times. The largest number of views was generated from professionals (624,770/994,494, 63.82%) followed by consumer videos (256,373/994,494, 25.78%) and news-based videos (113,351/994,494, 11.40%). There was a statistically significant difference between the number of views of consumer videos and professional videos (*W*=622, *P*=.04) but not between consumer videos and news videos (*W*=254, *P*=.16) or between news videos and professional videos (*W*=285.5, *P*=.54). Pairwise comparison of the lengths of videos between each category found no statistically significant differences (consumer vs news: *W*=350.5, *P*=.81; consumer vs professional: *W*=779.5, *P*=.58; news vs professional: *W*=276.5, *P*=.44). We found no correlation between log-transformed lengths and log-transformed views (Spearman’s rho =−.0066, *P*=.95).

The frequency of diabetic retinopathy videos by content and source are displayed in Table 2. In over one-third of the videos, a male was providing information (36/98, 37%). A purpose of the majority of videos was to provide information (59/98, 60%), and most of the videos showed or mentioned retinopathy in general (75/98, 77%). Smaller numbers offered information about specific types of retinopathy, namely proliferative (26/98, 27%) and nonproliferative (17/98, 17%). Other eye complications related to diabetes were rarely mentioned, with macular degeneration and cataracts being mentioned in fewer than 10% of the videos. The majority of videos (56/98, 57%) mentioned vision loss and blindness, but under half mentioned the importance of screening (40/98, 41%). Symptoms (48/98, 49%) and treatment (56/98, 57%) were frequently mentioned, but prevention for retinopathy was mentioned in only one-third of the videos (33/98, 34%).

The odds ratio of categories of sources of YouTube videos as compared to consumer-generated videos for each content category is presented in Table 3. Findings indicate that, when compared with consumer-generated videos with no people presenting information, news videos were 6.55 times more likely to have a male presenting information (95% CI 1.17-36.61) and 9 times more likely to have males and females both presenting information (95% CI 1.03-78.57). Similarly, when compared with consumer-generated videos with no people presenting information, professional videos were 4.64 times more likely to have males presenting information (95% CI 1.40-15.32) and 7 times more likely than professional videos to have males and females presenting information (95% CI 1.36-36.01). Compared with consumer-generated videos, professional videos were 5.57 times more likely to mention that diabetic retinopathy can go unnoticed (95% CI 1.59-26.15).

**Table 1:** Length of videos and the number of views of 98 diabetic retinopathy–related videos in English.

		Video length (in minutes)				Number of views				
	n	Mean (SE)	Median	Range	95% CI	Mean (SE)	Median	Range	95% CI	Total (%)
Consumer	42	10.24 (3.0)	2.90	0.25-97.60	4.04-16.44	6104 (1578)	3992	1728-68,540	2916-9292	256,373 (26)
News	16	6.49 (2.72)	2.23	0.59-44.48	0.69-12.29	7084 (1211)	6122	1848-17,760	4503-9666	113,351 (11)
Professional	40	8.26 (2.88)	3.98	0.42-113.02	2.45-14.08	15,620 (3422)	6194	1758-119,100	8698-22,540	624,770 (63)
Overall	98	8.82 (1.81)	3.24	0.25-113	5.23-12.41	10,148 (1620)	5169	1728-119,100	6933-13,363	994,494 (100)

**Table 2:** Frequency count of 98 diabetic retinopathy–related videos in English by their sources and contents.

		Source category of videos			
Content category		Consumer (n=42) n (%)	News (n=16) n (%)	Professional (n=40) n (%)	Total (N=98) n (%)
**Gender of person providing information in the video**					
	No people featured	18 (43)	2 (13)	6 (15)	26 (27)
	Man featured	11 (26)	8 (50)	17 (43)	36 (37)
	Woman featured	10 (24)	3 (19)	10 (25)	23 (24)
	Both featured	3 (7)	3 (19)	7 (18)	13 (13)
**Purpose of the video was to provide information about diabetic retinopathy**					
	No	21 (50)	5 (31)	13 (33)	39 (40)
	Yes	21 (50)	11 (69)	27 (68)	59 (60)
**Shows or mentions retinopathy**					
	No	11 (26)	4 (25)	8 (20)	23 (24)
	Yes	31 (74)	12 (75)	32 (80)	75 (77)
**Shows or mentions proliferative retinopathy**					
	No	29 (69)	15 (94)	28 (70)	72 (74)
	Yes	13 (31)	1 (6)	12 (30)	26 (27)
**Shows or mentions nonproliferative retinopathy**					
	No	34 (81)	16 (100)	31 (78)	81 (83)
	Yes	8 (19)	0 (0)	9 (23)	17 (17)
**Mentions screening**					
	No	27 (64)	8 (50)	23 (58)	58 (59)
	Yes	15 (36)	8 (50)	17 (43)	40 (41)
**Mentions macular degeneration**					
	No	40 (95)	14 (88)	36 (90)	90 (92)
	Yes	2 (5)	2 (13)	4 (10)	8 (8)
**Mentions vision loss or blindness**					
	No	21 (50)	5 (31)	16 (40)	42 (43)
	Yes	21 (50)	11 (69)	24 (60)	56 (57)
**Mentions cataract**					
	No	39 (93)	16 (100)	37 (93)	92 (94)
	Yes	3 (7)	0 (0)	3 (8)	6 (6)
**Mentions pain (if any)**					
	No	39 (93)	16 (100)	39 (98)	94 (96)
	Yes	3 (7)	0 (0)	1 (3)	4 (4)
**Mentions anxiety or fear of diagnosis or screening**					
	No	40 (95)	15 (94)	38 (95)	93 (95)
	Yes	2 (5)	1 (6)	2 (5)	5 (5)
**Mentions control of diabetes**					
	No	25 (60)	5 (31)	17 (43)	47 (48)
	Yes	17 (41)	11 (69)	23 (58)	51 (52)
**Mentions symptoms (if any)**					
	No	24 (57)	6 (38)	20 (50)	50 (51)
	Yes	18 (43)	10 (63)	20 (50)	48 (49)
**Mentions treatment (if any)**					
	No	17 (41)	9 (56)	16 (40)	42 (43)
	Yes	25 (60)	7 (44)	24 (60)	56 (57)
**Mentions prevention for retinopathy**					
	No	30 (71)	8 (50)	27 (68)	65 (66)
	Yes	12 (29)	8 (50)	13 (33)	33 (33)
**Mentions that it can go unnoticed**					
	No	39 (93)	13 (81)	28 (70)	80 (82)
	Yes	3 (7)	3 (19)	12 (30)	18 (18)
**Mentions retinal detachment**					
	No	36 (86)	13 (81)	29 (73)	78 (80)
	Yes	6 (14)	3 (19)	11 (28)	20 (20)

**Table 3:** The odds ratios of news and professional videos carrying contents pertinent to certain content compared with consumer-generated videos.

Content category		Odds ratio (95% CI)	*P* value
**Gender of person providing information in the video (reference group: no people featured; reference group: consumer videos)**			
	News: man featured	6.55 (1.17-36.61)	.032
	News: woman featured	2.70 (0.38-18.96)	.318
	News: both featured	9.00 (1.03-78.57)	.047
	Professional: man featured	4.64 (1.40-15.32)	.012
	Professional: woman featured	3.00 (0.84-10.72)	.091
	Professional: both featured	7.00 (1.36-36.01)	.020
**Purpose of the video was to provide information about diabetic retinopathy**			
	News	2.20 (0.65-7.44)	.205
	Professional	2.08 (0.85-5.09)	.110
**Shows or mentions retinopathy**			
	News	1.06 (0.28-4.00)	.926
	Professional	1.42 (0.50-4.00)	.508
**Shows or mentions proliferative retinopathy**			
	News	0.15 (0.02-1.25)	.079
	Professional	0.96 (0.37-2.45)	.925
**Shows or mentions nonproliferative retinopathy**			
	News^a^	—	—
	Professional	1.23 (0.42-3.60)	.700
**Mentions screening**			
	News	1.80 (0.56-5.77)	.323
	Professional	1.33 (0.55-3.24)	.529
**Mentions macular degeneration**			
	News	2.86 (0.37-22.24)	.316
	Professional	2.22 (0.38-12.87)	.373
**Mentions vision loss or blindness**			
	News	2.20 (0.65-7.44)	.205
	Professional	1.50 (0.63-3.60)	.364
**Mentions cataract**			
	News^a^	—	—
	Professional	1.05 (0.20-5.56)	.951
**Mentions pain (if any)**			
	News^a^	—	—
	Professional	0.33 (0.03-3.35)	.350
**Mentions anxiety or fear of diagnosis or screening**			
	News	1.33 (0.11-15.81)	.82
	Professional	1.05 (0.14-7.85)	.96
**Mentions control of diabetes**			
	News	3.24 (0.95-11.00)	.060
	Professional	1.99 (0.83-4.79)	.125
**Mentions symptoms (if any)**			
	News	2.22 (0.68-7.25)	.186
	Professional	1.33 (0.56-3.18)	.517
**Mentions treatment (if any)**			
	News	0.53 (0.17-1.69)	.284
	Professional	1.02 (0.42-2.47)	.965
**Mentions prevention for retinopathy**			
	News	2.50 (0.76-8.19)	.130
	Professional	1.20 (0.47-3.09)	.699
**Mentions that it can go unnoticed**			
	News	3.00 (0.54-16.74)	.210
	Professional	5.57 (1.44-21.60)	.013
**Mentions retinal detachment**			
	News	1.38 (0.30-6.36)	.676
	Professional	2.28 (0.75-6.90)	.146

^a^If all videos belong to a particular category of source of video, then we cannot calculate the odds ratio and the standard error will not be meaningful.

## Discussion

### Principal Findings

To our knowledge, this is the first study to describe the content of YouTube videos related to diabetic retinopathy. The importance of this eye disease is highlighted by the personal consequences for individuals affected [3], the large increase in incidence projected in the coming decades [3], and by the racial/ethnic disparities in recommended screening [5]. The availability of eye care professionals is unequally distributed throughout the United States [12,13] and individuals with lower levels of education and income have been shown to be less likely to have had an annual eye care visit [14,15]. Audiovisual communications such as YouTube videos are, therefore, a potentially effective approach for helping high-risk individuals make informed decisions about diabetic retinopathy screening.

With pervasive use of mobile technology, efforts using innovative communication methods are emerging. Systematic reviews of mHealth interventions for facilitating self-management of long-term illness [16] and preventive health care [17] have yielded equivocal findings. Nevertheless, there is some evidence for the value of mHealth interventions, for example, to promote lifestyle modifications associated with development of diabetes [18], and digital approaches to diabetic retinopathy screening are emerging as a way to increase access to preventive care [19]. While communication media such as YouTube have the potential to increase awareness and interest about preventing vision loss caused by diabetic retinopathy and assist individuals in making informed choices about screening and preventive care, our data show that more than 80% of the most widely viewed diabetic retinopathy videos did not address the asymptomatic nature of the disease; only about one-third mentioned prevention, and only 58 of the 98 videos mentioned screening. Thus, while digital media such as YouTube have the potential to contribute to diabetic retinopathy prevention, to realize this will require finding ways to reach consumers, especially racial/ethnic minority groups and those with lower levels of income and education, with communications that not only reach their intended audience but contain clear, accurate, and culturally sensitive messages about the importance of early detection and treatment.

### Limitations

This study was limited by the cross-sectional design, the inability to delineate the country of origin of each video, and the fact that it was limited to those videos with contents in English. In addition, the sample size was relatively small and the cut-off point of 100 videos was arbitrary. Despite these limitations, this study begins to fill a gap in the literature related to diabetic retinopathy and YouTube.

### Conclusions

Future research is needed to identify aspects of YouTube videos that attract viewer attention and best practices for using this medium for increasing diabetic retinopathy screening among people with diabetes.

## Abbreviations

CDC: Centers for Disease Control and Prevention
NEI: National Eye Institute
